# Performance Enhancement of Lightweight PLA Parts Printed by FFF Using Taguchi–GRA Method

**DOI:** 10.3390/polym17172413

**Published:** 2025-09-05

**Authors:** Oğuz Tunçel, Çağlar Kahya

**Affiliations:** 1 Department of Mechanical Engineering, Faculty of Engineering, Siirt University, Siirt 56100, Türkiye; 2 Department of Mechanical Engineering, Faculty of Engineering, Bursa Uludağ University, Bursa 16059, Türkiye; ckahya@uludag.edu.tr

**Keywords:** lightweight PLA, FFF, Taguchi approach, GRA, parameter optimization

## Abstract

Lightweight PLA (LW-PLA) filaments enable material-saving designs in fused filament fabrication (FFF), yet optimizing their mechanical performance remains challenging due to temperature-sensitive foaming behavior. This study aims to enhance the structural strength and material efficiency of LW-PLA parts using a multi-objective statistical approach. Four key process parameters—infill density (Id), material flow rate (Mf), wall line count (Wlc), and infill pattern (Ip)—were systematically varied using a Taguchi L_16_ orthogonal array. Tensile strength (Ts), flexural strength (Fs), and material consumption (Mc) were selected as the critical response metrics. Grey Relational Analysis (GRA) was used to aggregate these responses into a single performance index, and ANOVA determined each factor’s contribution. The optimal combination of 60% infill density, 70% material flow, 4 wall lines, and line infill pattern yielded a 9.02% improvement in the overall performance index compared to the baseline, with corresponding Ts and Fs values of 13.58 MPa and 20.51 MPa. Mf and Wlc were the most influential parameters on mechanical behavior, while Id mainly affected Mc. These findings confirm that integrating Taguchi and GRA enables effective parameter tuning for LW-PLA, balancing strength and efficiency. This work contributes to the development of lightweight, high-performance parts suitable for functional applications such as UAVs and prototyping.

## 1. Introduction

Fused filament fabrication (FFF) is one of the most widely used additive manufacturing (AM) technologies due to its low cost, material diversity, and ease of use [1,2]. This technique enables the production of objects with complex geometries by laying down layers of melted thermoplastic filament. In recent years, especially with composites and nanomaterials, the mechanical properties of FFF’s parts have significantly improved [3]. However, the products often exhibit non-isotropic mechanical behavior due to weak interlayer bonding. Therefore, optimizing the manufacturing parameters is critical to improving print quality and structural strength [4,5].

Polylactic acid (PLA) is one of FFF’s most commonly used materials due to its low melting point, biodegradability, and environmentally friendly nature [6]. Lightweight PLA (LW-PLA), a modified variant with reduced density, offers significant advantages in terms of material efficiency by minimizing weight and shortening print times. This makes it particularly suitable for applications where reducing part mass is critical. However, interlayer bonding strongly influences mechanical performance, which depends on key manufacturing parameters such as infill density and material flow rate [7,8,9]. Therefore, carefully optimizing these parameters is essential to balance lightweight design and structural reliability [10].

The mechanical behavior of PLA-based parts in FFF is highly sensitive to process parameters, particularly when lightweight structures are targeted. Studies show that parameters such as infill density, wall line count, extrusion temperature, and printing speed substantially influence tensile and flexural performance, part mass, and production efficiency [11,12,13]. In the context of LW-PLA, which is inherently lower in density, these parameters become even more critical for ensuring structural integrity. For instance, making LW-PLA is ideal for applications requiring a high strength-to-weight ratio [14,15]. Furthermore, careful tuning of extrusion parameters can enhance interlayer bonding, thereby mitigating the inherent anisotropy of FFF-printed parts and extending their usability in functional or load-bearing applications [16,17]. As such, optimization becomes a key strategy for leveraging the material-saving benefits of LW-PLA without compromising mechanical performance.

Identifying the optimal combination of FFF process parameters is challenging due to the complex, nonlinear interactions among variables such as infill density, material flow, wall count, and infill pattern. Traditional trial-and-error approaches are time-consuming and often fail to capture the multifactorial nature of AM processes. To overcome these limitations, the Taguchi method provides a statistically robust and efficient design of experiments (DoE) framework that reduces the number of tests while retaining analytical clarity [18,19,20]. However, single-objective optimization is insufficient in applications involving competing performance goals, such as maximizing mechanical strength while minimizing material usage. Grey relational analysis (GRA), when integrated with the Taguchi method, enables the simultaneous evaluation of multiple responses, converting them into a single performance index known as the Grey relational grade (GRG) [21,22]. This combined approach has been successfully applied in FFF studies using PLA to determine optimal parameter configurations for dimensional accuracy and material performance [23,24], forming this study’s methodological basis.

Recent advances in using LW-PLA in FFF have demonstrated the material’s potential for achieving lightweight structures without severely compromising mechanical integrity. Park and Lee [25] utilized dual-nozzle FFF to produce PLA/LW-PLA composite structures, finding that higher nozzle temperatures (230–240 °C) and full infill density significantly enhanced toughness by up to 246.5% due to improved pore compression effects. Similarly, Kanani et al. [26] showed that adjusting deposition temperature and extrusion multiplier in material extrusion (MEX) printing enabled control over foaming behavior and density, highlighting a trade-off between stiffness and weight. Damanpack et al. [27] further confirmed that printing temperature and flow rate directly influences pore size and mechanical response, providing an empirical model to optimize porous LW-PLA performance. Nofar et al. [28] reviewed strategies for combining foaming and 3D printing processes, identifying slicing settings, material saturation, and blowing agents as key to lightweight thermoplastic structures. Tao et al. [29] demonstrated that PLA lattice structures filled with rigid polyurethane foam significantly improved energy absorption, suggesting potential for hybrid LW-PLA systems in load-bearing applications. Complementing these, Prajapati et al. [30] introduced a hybrid FFF process that integrates foam-filling of closed-cell TPU lattices with polyurethane foam, enhancing stiffness, damping, and energy dissipation without added post-processing, offering a promising template for functional multi-material LW-PLA composites.

The present study aims to improve the mechanical performance and material efficiency of LW-PLA parts fabricated by FFF through a multi-response optimization approach. Four key process parameters—infill density (Id), material flow (Mf), wall line count (Wlc), and infill pattern (Ip)—were selected and investigated using a Taguchi L_16_ orthogonal array. The experimental results were evaluated using signal-to-noise (S/N) ratios and analysis of variance (ANOVA), while the optimal parameter combination was identified through GRA. Although the Taguchi–GRA method has been extensively applied to standard PLA in previous studies [12,31], its application to LW-PLA remains limited. LW-PLA exhibits temperature-dependent foaming behavior during extrusion, which introduces additional complexity in controlling mechanical properties and dimensional accuracy. Therefore, this study provides a novel contribution by systematically applying statistical optimization techniques to foamed PLA structures, revealing process–performance relationships that differ significantly from solid PLA and offering practical insights for lightweight, material-efficient AM. In addition, comparative tests were performed with conventional PLA under the optimized condition to highlight the distinct process–performance characteristics of LW-PLA. The results confirmed that LW-PLA provides significant weight reduction and improved flexural behavior compared to PLA, emphasizing the necessity of parameter-specific optimization for foamed PLA structures.

## 2. Materials and Methods

### 2.1. Materials

A LW-PLA filament developed by Filameon (Kayseri, Türkiye) was used in this study. This material features a foaming structure at elevated temperatures, enabling significant weight reduction during printing. The nominal diameter of the filament was 1.75 mm. Its low-density structure and process-dependent foaming behavior make it particularly suitable for applications where weight saving is critical, such as unmanned aerial vehicle (UAV) fuselages, model aircraft, RC components, and mold or prototype fabrication. The physical properties provided by the manufacturer are summarized in Table 1.

### 2.2. Specimen Design and 3D Printing Setup

**Figure 1:**
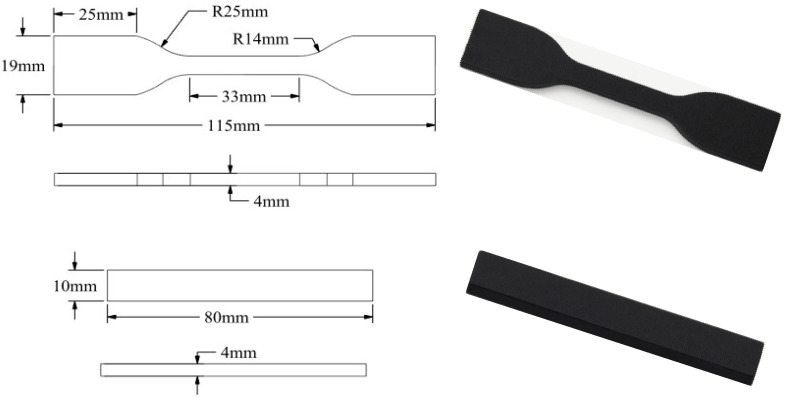
CAD model of an ASTM D638 Type IV tensile test specimen and flexural test specimens.

The tensile and flexural specimens were designed following ASTM D638 Type IV and ISO 178 standards [32,33]. The models were prepared in SolidWorks 2022 and sliced using Ultimaker Cura 5.10.0. Printing was performed using a Creality Ender-3 S1 Pro (Shenzen, China) FFF 3D printer. The printing process was conducted under ambient conditions without an enclosure. All specimens were printed in a flat orientation on the XY plane. A ±45° raster angle was used throughout the printing process, and the toolpath strategy was kept constant across all experimental runs to ensure comparability of results. A visual representation of the printed specimens is given in Figure 1.

### 2.3. Experimental Design

To investigate the effect of process parameters on mechanical strength and material consumption of LW-PLA parts fabricated via FFF, four key parameters were selected: infill density (Id), material flow (Mf), wall line count (Wlc), and infill pattern (Ip). Each parameter was examined at four levels, as presented in Table 2. These parameter ranges were determined through preliminary trial prints and practical evaluations informed by the foaming sensitivity and mechanical behavior of LW-PLA. Although the general influence of parameters such as infill density, material flow, and wall line count on mechanical strength or material usage can be qualitatively anticipated, their combined effects and relative statistical significance are highly material-dependent. In LW-PLA, foaming-sensitive extrusion introduces non-linear interactions that cannot be reliably predicted without systematic experimentation. Therefore, the selected parameters were chosen to experimentally validate and quantify their contributions under LW-PLA conditions. Id values below 30% resulted in structurally weak specimens with insufficient interlayer bonding, while values above 60% reduced the effectiveness of the lightweight design. Therefore, a range of 30–60% was selected to maintain a balance between mass reduction and mechanical performance. Mf was varied between 55% and 70% to ensure consistent extrusion; lower values caused under-extrusion, whereas higher values led to over-deposition and dimensional inaccuracies. Wlc was adjusted from 1 to 4 to investigate the role of shell thickness and the bonding quality between outer and inner walls. Lastly, four widely adopted Ip types—line, grid, gyroid, and zigzag—were selected to evaluate their impact on internal structural support and material efficiency.

**Figure 2:**
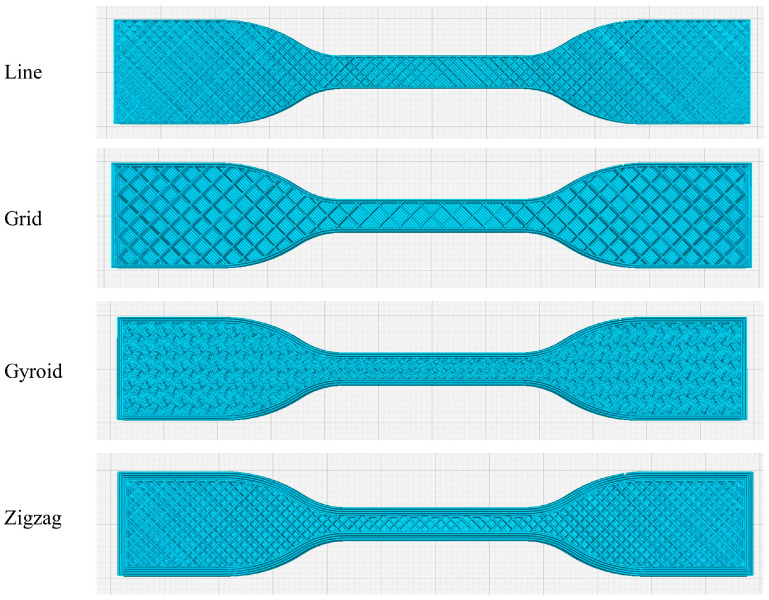
Schematic illustrations of the four infill patterns considered in this study, as generated in Ultimaker Cura 5.10.0: Line, Grid, Gyroid, and Zigzag.

The actual geometries of the four infill patterns generated in Ultimaker Cura 5.10.0 are presented in Figure 2 to ensure clarity and reproducibility. Providing these schematic illustrations allows readers to precisely identify the internal structures used in this study and eliminates potential ambiguities that may arise from slicer-dependent variations, thereby ensuring that the results can be consistently replicated in future research.

A Taguchi orthogonal array L_16_ (4^4^) design was employed to efficiently reduce the number of experimental runs while still capturing the interactions among variables. This experimental layout enabled the examination of 16 unique parameter combinations; each replicated three times to ensure result consistency. All other printing conditions were held constant throughout the study to isolate the effects of the four varying parameters. These fixed parameters—including nozzle diameter, temperature settings, print speed, raster angle, and fan speed—are detailed in Table 3.

Preliminary printing trials also confirmed the temperature-dependent foaming behavior of LW-PLA. At nozzle temperatures below 230 °C, specimens appeared darker (blackish) and exhibited wall–infill bonding defects, indicating insufficient foaming. At 230 °C, however, the parts turned grayish and no bonding defects were observed, confirming that foaming was effectively activated under the selected condition.

### 2.4. Mechanical Testing

**Figure 3:**
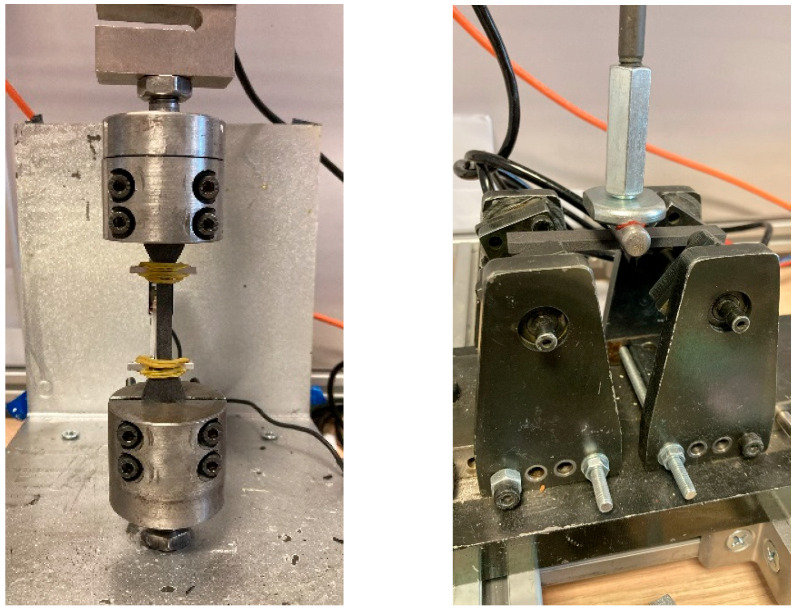
Representative images of the printed specimens and the mechanical testing setup.

Mechanical characterization was performed to evaluate the printed specimens’ tensile strength (Ts), flexural strength (Fs), and material consumption (Mc). Tensile and flexural tests were carried out using a custom-built tabletop testing device (Figure 3) with a 2 kN load capacity, by ASTM D638 Type IV and ISO 178 standards, respectively; the flexural test employed a three-point bending setup with a span-to-depth ratio of 16:1 [34]. In both tests, the loading speed was set to 5 mm/min.

In total, 48 tensile specimens (16 conditions × 3 replicates) and 48 flexural specimens (16 conditions × 3 replicates) were tested. Three identical specimens were prepared for each experimental condition, and the average values and standard deviations were reported. Before mechanical testing, the mass of each specimen was measured using a precision digital scale (±0.01 g accuracy) to quantify Mc. Mc was considered an important optimization objective because it directly reflects the material-saving capability of LW-PLA, which is one of the main motivations for using this filament in lightweight structural applications.

### 2.5. Statistical and Multi-Objective Optimization Methods

This study used statistical and decision-making techniques to optimize three competing output responses—Ts, Fs, and Mc. The methodology integrated the Taguchi design of experiments, S/N analysis, GRA, and ANOVA.

#### 2.5.1. Signal-to-Noise Ratio (S/N)

S/N ratio analysis was first performed to assess the robustness of each response under varying process parameters. The following formulas were used for different response objectives.

For larger-is-better characteristics (Ts and Fs) [22]:

(1) $$\eta \;=\;-10\log\left(\frac{1}{n}\sum_{i=1}^{n}\frac{1}{y_{i}^{2}}\right)$$

For smaller-is-better characteristics (Mc) [35]:

(2) $$\eta \;=\;-10\log\left(\frac{1}{n}\sum_{i=1}^{n}y_{i}^{2}\right)$$

where $y_{i}$ is the observed value and n = 3 is the number of replications.

#### 2.5.2. Grey Relational Analysis (GRA)

The normalized S/N ratios were transformed into a single Grey Relational Grade (GRG) through GRA to enable multi-response optimization. Normalization was conducted based on the performance goal.

For larger-is-better [36]:

(3) $$x_{i}^{*}=\frac{y_{i}\;-\;\min\left(y\right)}{\max\left(y\right)\;-\;\min\left(y\right)}$$

For smaller-is-better [37]:

(4) $$x_{i}^{*}=\frac{\max\left(y\right)\;-\;y_{i}}{\max\left(y\right)\;-\;\min\left(y\right)}$$

Then, the Grey Relational Coefficient (GRC) was calculated as [38]:

(5) $$\xi_{i}=\frac{\Delta_{\min}\;+\;\zeta {.\Delta }_{\;\max}}{\Delta_{0i}\;+\;\zeta {.\Delta }_{\;\max}}$$

where Δ_i_ = ∣x_0_^∗^ − x_i_^∗^∣, Δ_min,_ and Δ_max_ are the minimum and maximum deviations, and ζ ∈ [0, 1] is the distinguishing coefficient (typically 0.5).

Finally, the GRG was computed as [39]:

(6) $$\gamma_{i}\;=\;\frac{1}{m}\sum_{j=1}^{m}{w_{f}.\xi }_{\;i\;j}$$

where m is the number of responses, and $w_{f}$ is the weight factor.

#### 2.5.3. Analysis of Variance (ANOVA)

ANOVA was applied to the individual S/N ratios and the GRG values to determine each process parameter’s statistical significance and contribution ratios. The F-ratio and corresponding *p*-values were calculated for each factor, and factors with *p* < 0.05 were considered statistically significant [40]. The delta (Δ) values—representing the difference between the maximum and minimum response means—were also used to rank parameter influence.

All statistical calculations, optimization procedures, and visual analyses—including main effect plots and GRG-based rankings—were conducted using Minitab 20.3 software.

## 3. Results and Discussions

Table 4 presents the experimental results obtained from 16 different parameter combinations, revealing considerable variation across all response metrics. Ts ranged from 5.13 MPa to 12.98 MPa, while Fs varied between 10.10 MPa and 20.24 MPa. Mc also exhibited a broad distribution from 2.80 g to 5.51 g. These preliminary results indicate that the selected process parameters notably affect mechanical properties and filament usage. A more detailed examination of parameter influences is provided in the subsequent sections through Taguchi-based analysis, ANOVA, and GRA.

### 3.1. Tensile Strength Analysis

The Ts results and corresponding S/N ratio analysis are summarized in Table 5 to evaluate the influence of each process parameter. Among the 16 experimental trials, the highest Ts value of 12.98 MPa was obtained under the parameter combination Id_1_–Mf_4_–Wlc_4_–Ip_4_, where Id was at its lowest level, but Mf, Wlc and Ip were set to their highest levels. In contrast, the lowest Ts (5.13 MPa) was recorded in Exp.1 (Id_1_–Mf_1_–Wlc_1_–Ip_1_), where all parameters were at their minimum settings. The S/N analysis revealed that Wlc had the most significant impact on Ts with a delta of 4.69, followed by Mf (Δ = 3.05) and Id (Δ = 2.17). The Ip exhibited the least effect with a delta of 0.58. According to the Taguchi method, the theoretical optimum parameter levels for maximizing Ts were identified as Id_4_–Mf_4_–Wlc_4_–Ip_4_, suggesting that high infill, maximum flow rate, increased shell thickness, and a zigzag pattern collectively contribute to improved tensile behavior. Under this optimum setting (Id_4_–Mf_4_–Wlc_4_–Ip_4_), a Ts value of 14.72 MPa was achieved, corresponding to a 13.41% improvement compared to the highest experimental value (12.98 MPa).

**Figure 4:**
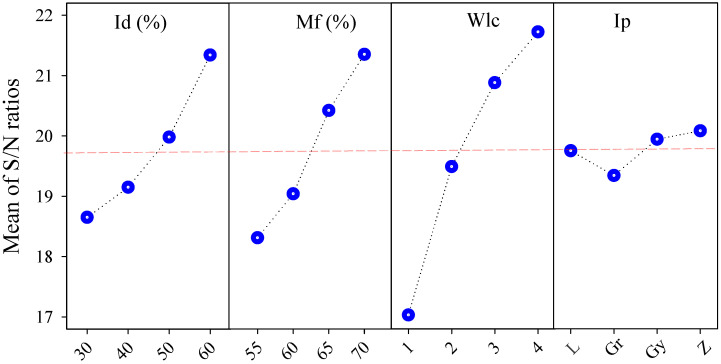
S/N ratio values for the tensile strength based on infill density, material flow, wall line count, and infill pattern.

Figure 4 illustrates the main effects plot for the S/N ratios of Ts, clearly visualizing the influence of each process parameter across its levels. A consistent upward trend is observed with increasing levels of Id, Mf, and Wlc, confirming their substantial positive impact on Ts. The S/N ratio increases from 18.65 dB at Id_1_ to 21.34 dB at Id_4_, reflecting an approximately 14.4% improvement. Similarly, Mf shows a rise from 18.31 dB (Mf_1_) to 21.35 dB (Mf_4_), and Wlc from 17.03 dB (Wlc_1_) to 21.72 dB (Wlc_4_), indicating improvements of 16.6% and 27.6%, respectively. These trends support the previous interpretation that higher shell thickness and extrusion volume enhance interlayer bonding, thereby increasing mechanical resistance [41]. Conversely, the Ip shows a relatively minor effect with a narrow S/N range of 0.74 dB, suggesting that mechanical strength is more sensitive to volumetric and geometric reinforcement than the pathing strategy. These findings validate the statistical ranking presented in Table 5 and highlight Wlc and Mf as dominant parameters for optimizing Ts in LW-PLA parts, which can be attributed to improved interlayer adhesion and localized foaming expansion caused by higher Mf, as well as increased shell integrity and reduced porosity associated with higher Wlc.

Table 6 presents the ANOVA results for Ts, confirming the statistical significance of the examined process parameters. Wlc emerged as the most influential factor, with the highest contribution ratio of 54.92% (*p* = 0.005), followed by Mf at 24.82% (*p* = 0.016) and Id at 16.66% (*p* = 0.027). The Ip, with a contribution of only 2.45% (*p* = 0.276), had a negligible effect. These findings align with the S/N analysis results and validate the dominance of Wlc and Mf in determining Ts. The high R^2^ value of 98.85% indicates a strong correlation between the experimental data and the statistical model.

### 3.2. Flexural Strength Analysis

**Figure 5:**
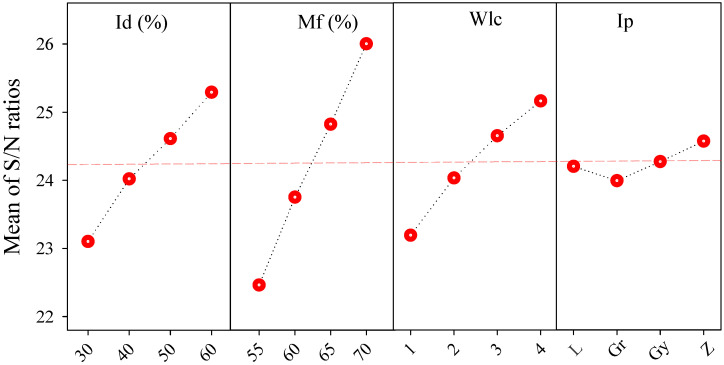
S/N ratio values for the flexural strength based on infill density, material flow, wall line count, and infill pattern.

The S/N ratio results for Fs are presented in Table 7. Among all trials, the highest Fs value (20.24 MPa) was obtained in Exp. 16, while the lowest (10.10 MPa) occurred in Exp. 1, corresponding to a 100% increase. Based on delta values, Mf (Δ = 3.55 dB) had the most pronounced effect, followed by Id (Δ = 2.19 dB), Wlc (Δ = 1.97 dB), and Ip (Δ = 0.58 dB). According to the Taguchi method, the optimal level combination for maximizing Fs was Id_4_–Mf_4_–Wlc_4_–Ip_4_. Under this optimum setting, an Fs of 21.83 MPa was achieved, representing a 7.86% improvement compared to the highest experimental value of 20.24 MPa. The improvements are mainly attributed to enhanced rigidity and interlayer fusion at higher flow and density settings [42]. These trends are consistent with Figure 5 and are further supported by statistical significance analysis in the subsequent section.

Figure 5 clearly illustrates that Mf had the most significant effect on Fs, with the S/N ratio increasing from 22.46 dB (Mf_1_) to 26.00 dB (Mf_4_), corresponding to a 15.8% rise. Similarly, Id showed a gradual increase from 23.10 dB to 25.29 dB, while Wlc exhibited a moderate impact with values ranging from 23.19 dB to 25.16 dB. Ip had minimal influence, showing only a 0.58 dB variation across levels. These findings confirm that enhancing flow rate and infill ratio improves interlayer bonding and rigidity, whereas the internal pattern contributes marginally to flexural performance. The parameter ranking from S/N analysis (Table 7) aligns closely with these graphical trends, as the increase in Id and Mf enhances cross-sectional stiffness and deposition quality, while higher Wlc contributes to outer wall reinforcement that redirects bending stress away from the internal structure.

Table 8 presents the ANOVA results for Fs. The most influential parameter was Mf, contributing 62.19% with a highly significant *p*-value (*p* = 0.001). It was followed by Id and Wlc, contributing 19.96% (*p* = 0.004) and 15.69% (*p* = 0.006), respectively. The effect of Ip was minor and statistically insignificant, with a contribution of 1.79% (*p* = 0.111). The model demonstrated excellent fit, as indicated by a high R^2^ value of 99.64%, confirming the robustness of the analysis.

### 3.3. Material Consumption Analysis

Mc is a critical parameter in lightweight design, reflecting filament usage efficiency. Table 9 presents the S/N ratio results, where all values are negative due to the “smaller-is-better” characteristic of the response. Among the investigated parameters, Id exhibited the most significant influence on Mc, with a delta value of 2.74 dB, followed closely by Mf with 2.60 dB. Wlc showed moderate impact (Δ = 1.38 dB), whereas Ip had the most negligible effect (Δ = 0.39 dB). According to the Taguchi method, the optimal configuration for minimizing Mc was Id_1_–Mf_1_–Wlc_1_–Ip_1_, corresponding to the lowest levels of all parameters. This indicates that reducing the volumetric fill and extrusion rate effectively decreases filament usage [43]. At this optimum setting, the lowest material consumption was recorded as 2.80 g (also seen in Exp.1).

**Figure 6:**
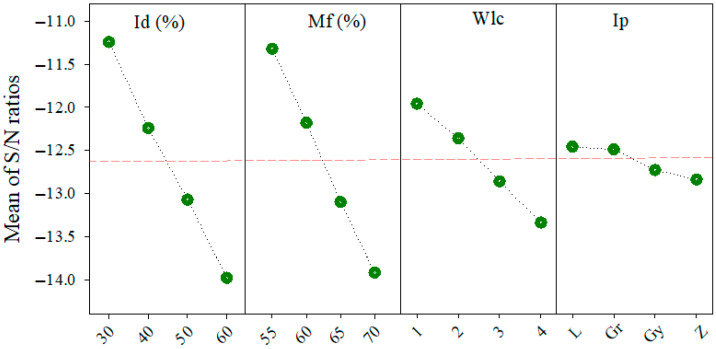
S/N ratio values for the material consumption are based on infill density, material flow, wall line count, and infill pattern.

Figure 6 further illustrates the trends in S/N ratios, reinforcing the dominant influence of Id and Mf on material usage. A steady decline in S/N ratios is observed as Id increases from 30% to 60%, indicating greater filament consumption. Similar behavior is seen for Mf, affirming the necessity of careful control over extrusion rate and infill percentage when aiming for lightweight structures.

ANOVA was performed and summarized in Table 10 to validate these findings statistically. The analysis confirms that Id was the most significant factor, contributing 46.51% to the overall variance (*p* = 0.001). Mf followed with 43.26% (*p* = 0.001), and Wlc contributed 8.84% (*p* = 0.013). In contrast, Ip was not statistically significant (*p* = 0.213) and accounted for only 1.01% of the variation. The model exhibited a very high coefficient of determination (R^2^ = 99.63%), indicating excellent goodness-of-fit and supporting the robustness of the statistical conclusions.

These results underline the dominant role of volumetric parameters—particularly Id and Mf—in dictating Mc in FFF-printed LW-PLA parts. They also highlight the potential for substantial filament savings through optimized parameter selection without compromising mechanical integrity, as further explored in the multi-objective optimization section, since both Id and Mf directly control the volumetric deposition rate and thus material usage. In contrast, the minimal effect of Ip indicates that deposition path strategy has less impact on mass compared to overall geometric fill.

### 3.4. GRA Analysis

In the multi-objective optimization stage, GRA integrated Ts, Fs, and Mc into a single GRG index. Since Ts and Fs are “larger-is-better” and Mc is “smaller-is-better,” S/N ratios were first normalized accordingly. Then, GRCs were calculated, and GRG values were derived for all 16 experiments. As shown in Table 11, the highest GRG value (0.820) was achieved in Exp. 4 (Id_1_–Mf_4_–Wlc_4_–Ip_4_), which corresponded to high values of Ts and Fs with moderate Mc. The lowest GRG (0.455) was observed in Exp. 11 (Id_3_–Mf_3_–Wlc_1_–Ip_2_), reflecting poor performance across all responses. The top-ranking configurations generally involved elevated levels of Mf and Id, indicating their significant contribution to mechanical performance. However, when all responses are considered together, Mf and Wlc emerged as the most influential factors in achieving balanced multi-response optimization.

Assigning appropriate weight factors to each output response is essential for enhancing the accuracy of multi-objective optimization using GRA. While some studies assume equal importance among responses (e.g., 33.33% for each), such assumptions can compromise result reliability. To address this, the weight factors for Ts, Fs, and Mc were objectively calculated based on their respective contribution ratios derived from ANOVA results. The normalized weight values were determined as 0.4202 for Ts, 0.3121 for Fs, and 0.2677 for Mc and were used in the weighted aggregation of the GRC to compute the GRG, as shown in Equation (7):

GRG = GRC_Ts_.0.4202 + GRC_Fs_.0.3121 + GRC_Mc_.0.2677 (7)

This approach ensures that each response contributes proportionally to the overall performance index, thereby increasing the robustness and sensitivity of the optimization results [44].

Table 12 shows the mean S/N ratios and delta values for each parameter concerning GRG. Among all parameters, Mf had the highest influence on overall performance with a delta value of 2.68 dB, followed closely by Wlc (Δ = 2.57 dB). Ip ranked third (Δ = 1.47 dB), while Id showed the least effect (Δ = 1.19 dB). These results indicate that Mf and Wlc dominate in achieving balanced multi-response optimization.

**Figure 7:**
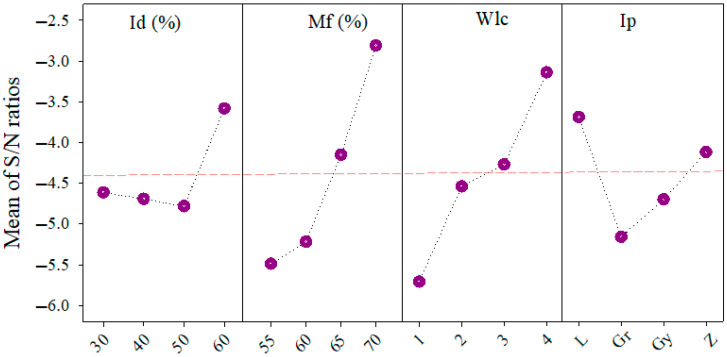
S/N ratio values for the GRG are based on infill density, material flow, wall line count, and infill pattern.

Figure 7 illustrates the main effects plot for the S/N ratios of GRG across the four input parameters. A consistent upward trend is observed with increasing levels of Mf and Wlc, indicating that higher Mf and more outer walls positively contribute to multi-objective performance. While Ip shows a non-monotonic trend with a peak at level 1, Id exhibits the lowest overall influence, with relatively flat S/N variation across levels. These graphical patterns align with the delta rankings in Table 12, reinforcing the dominant roles of Mf and Wlc in optimizing overall response quality.

Table 13 summarizes the ANOVA results for GRG. Among the process parameters, Mf had the highest statistical influence with a contribution of 45.10% (*p* = 0.032), indicating its dominant role in overall performance optimization. It was followed by Wlc (31.06%, *p* = 0.053) and Ip (12.97%, *p* = 0.156), while Id had the lowest contribution (7.37%, *p* = 0.279). Although not all parameters reached statistical significance (*p* < 0.05), the model demonstrated a strong fit, as reflected by a high R^2^ value of 99.49%. These results validate the S/N-based ranking and support the emphasis on volumetric parameters in multi-objective improvement, as Mf influences both mechanical strength and deposition stability through foaming-induced expansion and bonding continuity, while Wlc reduces structural anisotropy by reinforcing the outer walls that predominantly resist applied loads. This behavior, in contrast to conventional PLA studies, highlights how the foaming sensitivity of LW-PLA significantly alters the influence of flow rate and wall thickness on both mechanical and material performance.

Table 14 shows the improvement in GRG obtained by transitioning from the initial parameter setting (Id_1_–Mf_4_–Wlc_4_–Ip_4_) to the GRA-based optimal setting (Id_4_–Mf_4_–Wlc_4_–Ip_1_). The GRG value increased from 0.820 to 0.894, corresponding to an improvement of 0.074, or 9.02%. This enhancement confirms that the optimal parameter combination provides a more balanced trade-off between mechanical strength and material efficiency. While Ts and Fs showed noticeable improvements, Mc also increased due to higher Id and flow settings required to achieve enhanced strength. Moreover, the maximum error between the predicted and experimental GRG values was calculated as 2.72%, indicating strong predictive accuracy of the GRA model.

### 3.5. Comparison with Conventional PLA

To further clarify the advantages of LW-PLA, additional specimens were fabricated using standard PLA under the GRA-based optimal parameter set (Id_4_–Mf_4_–Wlc_4_–Ip_1_). The comparative results revealed distinct differences between the two materials. The PLA specimen exhibited a mass of 6.37 g, tensile strength of 17.81 MPa, and flexural strength of 16.19 MPa. In contrast, the LW-PLA counterpart demonstrated a lower mass of 5.46 g (corresponding to a 14.3% reduction), tensile strength of 13.58 MPa, and flexural strength of 20.51 MPa.

**Figure 8:**
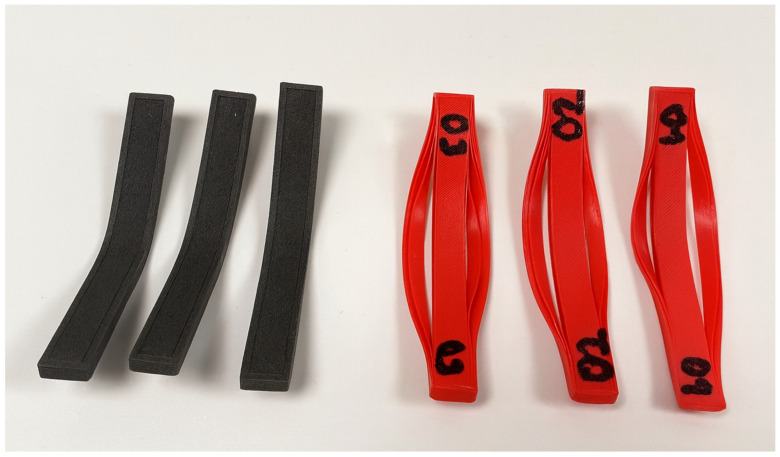
Representative flexural specimens of LW-PLA (**left**, black) and PLA (**right**, red) produced under the optimized parameter set (Id_4_–Mf_4_–Wlc_4_–Ip_1_).

These findings highlight the trade-offs between the two materials. While PLA provided higher tensile strength, LW-PLA offered substantial weight savings along with improved flexural resistance, making it more suitable for lightweight structural applications where bending performance and efficiency are prioritized. Furthermore, as illustrated in Figure 8, PLA specimens printed at the same optimized low material flow (Mf) occasionally suffered from incomplete bonding between the outer walls and internal regions, resulting in local defects. This indicates that parameter optimization cannot be directly extrapolated from conventional PLA to LW-PLA, as the foaming-sensitive behavior of LW-PLA introduces distinct process–performance interactions.

## 4. Conclusions

This study presented a multi-objective optimization of FFF process parameters for lightweight PLA parts, integrating the Taguchi method and GRA to enhance tensile and flexural strength while minimizing material consumption. The main findings are summarized as follows:

The Taguchi–GRA method identified the optimal parameter set as 60% Id, 70% Mf, 4 Wlc, and line Ip, which yielded the highest GRG value (0.894) and corresponding tensile and flexural strengths of 13.58 MPa and 20.51 MPa, respectively.

The optimized sample based on GRA exhibited a 9.02% improvement in GRG compared to the initial condition, confirming the effectiveness of the Taguchi–GRA integration for multi-response optimization.

ANOVA results showed that Mf and Wlc were the most influential parameters for mechanical performance, while Id predominantly affected Mc.

Validation experiments showed strong agreement with the predicted values from the GRA model, with a maximum error of 2.72%, demonstrating the reliability and practical relevance of the proposed optimization framework.

A comparative test with conventional PLA under the optimized condition confirmed the distinct advantages of LW-PLA. While PLA achieved higher tensile strength (17.81 MPa), LW-PLA exhibited superior flexural strength (20.51 MPa) with a 14.3% reduction in mass, highlighting its suitability for lightweight applications where efficiency and bending resistance are prioritized.

Future research could explore the long-term durability and thermal stability of optimized LW-PLA components under cyclic loading and environmental exposure, which are critical for functional applications such as UAVs and RC models. In addition, comparative investigations against other lightweight additive manufacturing strategies—such as multi-material combinations, core–shell structures, or reinforced laminates—would provide further insight into the relative advantages and limitations of single-material LW-PLA systems. Exploring closed-loop recycling or re-foaming strategies for LW-PLA could also enhance its applicability in sustainable manufacturing contexts.

## Figures and Tables

**Table 1:** Physical properties of LW-PLA filament.

	Value
Filament diameter	1.75 mm
Material type	LW-PLA
Density (unfoamed)	1.20 g/cm^3^
Tolerance	±0.03 mm
Recommended print temperature	195–260 °C
Bed temperature (optional)	45–65 °C
Foaming activation temperature	~230 °C
Tensile strength	30–32 MPa
Elongation at break	6–8%
MFI (210 °C/2.16 kg)	6
Heat resistance	55 °C
Manufacturer	Filameon (Türkiye)

**Table 2:** Three-dimensional printing process parameters and their levels.

Symbol	Parameters	Unit	Levels			
			1	2	3	4
Id	Infill density	%	30	40	50	60
Mf	Material flow	%	55	60	65	70
Wlc	Wall line count	-	1	2	3	4
Ip	Infill pattern	-	Line (L)	Grid (Gr)	Gyroid (Gy)	ZigZag (Z)

**Table 3:** Fixed 3D printing parameters.

Parameter	Unit	Value
Nozzle diameter	mm	0.4
Layer height	mm	0.2
Top and bottom layers	-	2
Print speed	mm/s	40
Nozzle temperature	°C	230
Table temperature	°C	60
Raster angle	°	45/−45
Fan speed	%	50
Retraction	-	Disable

**Table 4:** L_16_ (4^4^) orthogonal array for FFF and response values.

Exp. No.	Parameters				Results		
	Id (%)	Mf (%)	Wlc	Ip	Ts (MPa) ± sd	Fs (MPa) ± sd	Mc (g) ± sd
1	30	55	1	L	5.13 ± 0.11	10.10 ± 0.06	**2.80 ± 0.08**
2	30	60	2	Gr	7.67 ± 0.09	12.94 ± 0.16	3.35 ± 0.09
3	30	65	3	Gy	10.53 ± 0.18	16.07 ± 0.18	3.98 ± 0.11
4	30	70	4	Z	**12.98 ± 0.17**	19.84 ± 0.22	4.73 ± 0.11
5	40	55	2	Gy	7.36 ± 0.21	12.44 ± 0.14	3.47 ± 0.05
6	40	60	1	Z	6.20 ± 0.17	13.83 ± 0.14	3.67 ± 0.06
7	40	65	4	L	12.90 ± 0.18	18.98 ± 0.17	4.65 ± 0.07
8	40	70	3	Gr	11.46 ± 0.13	19.54 ± 0.02	4.72 ± 0.08
9	50	55	3	Z	10.51 ± 0.20	15.24 ± 0.10	4.13 ± 0.08
10	50	60	4	Gy	11.40 ± 0.12	17.63 ± 0.19	4.63 ± 0.10
11	50	65	1	Gr	7.27 ± 0.13	15.36 ± 0.12	4.36 ± 0.08
12	50	70	2	L	11.39 ± 0.13	20.23 ± 0.12	4.95 ± 0.10
13	60	55	4	Gr	11.55 ± 0.05	16.18 ± 0.18	4.57 ± 0.09
14	60	60	3	L	11.85 ± 0.11	17.80 ± 0.20	4.81 ± 0.09
15	60	65	2	Z	12.28 ± 0.05	19.61 ± 0.17	5.17 ± 0.09
16	60	70	1	Gy	11.01 ± 0.12	**20.24 ± 0.16**	5.51 ± 0.10
Min.	-	-	-	-	5.13	10.10	2.80
Max.	-	-	-	-	12.98	20.24	5.51
Median	-	-	-	-	7.85	10.13	2.71
Average	-	-	-	-	10.09	16.63	4.34

**Table 5:** Mean S/N ratios and ranking parameters of the tensile strength.

Level	Id	Mf	Wlc	Ip
1	18.65	18.31	17.03	19.75
2	19.15	19.04	19.49	19.34
3	19.98	20.42	20.88	19.94
4	**21.34**	**21.35**	**21.72**	**20.08**
Delta	2.68	3.05	4.69	0.74
∑ Delta	11.16			
Weight	42.02%			
Rank	3	2	1	4

**Table 6:** ANOVA table for tensile strength.

Source	DF	Adj SS	Adj MS	F-Value	*p*-Value	Contribution (%)
Id	3	16.616	5.205	14.479	0.027	16.66
Mf	3	23.263	7.754	21.570	0.016	24.82
Wlc	3	51.473	17.158	47.727	0.005	54.92
Ip	3	2.294	0.765	2.127	0.276	2.45
Error	3	1.078	0.359			1.15
Total	15					100
R^2^ = 98.85%						

**Table 7:** Mean S/N ratios and ranking parameters of the flexural strength.

Level	Id	Mf	Wlc	Ip
1	23.10	22.46	23.19	24.20
2	24.02	23.75	24.03	23.99
3	24.61	24.82	24.65	24.27
4	**25.29**	**26.00**	**25.16**	**24.57**
Delta	2.19	3.55	1.97	0.58
∑ Delta	8.29			
Weight	31.21%			
Rank	2	1	3	4

**Table 8:** ANOVA table for flexural strength.

Source	DF	Adj SS	Adj MS	F-Value	*p*-Value	Contribution (%)
Id	3	29.389	9.796	54.840	0.004	19.96
Mf	3	91.573	30.524	170.876	0.001	62.19
Wlc	3	23.110	7.703	43.124	0.006	15.69
Ip	3	2.641	0.880	4.929	0.111	1.79
Error	3	0.536	0.179			0.36
Total	15					100
R^2^ = 99.64%						

**Table 9:** Mean S/N ratios and ranking parameters of the material consumption.

Level	Id	Mf	Wlc	Ip
1	**−11.24**	−**11.32**	**−11.96**	**−12.46**
2	−12.24	−12.18	−12.36	−12.49
3	−13.07	−13.10	−12.86	−12.73
4	−13.98	−13.92	−13.34	−12.84
Delta	2.74	2.60	1.38	0.39
∑ Delta	7.11			
Weight	26.77%			
Rank	1	2	3	4

**Table 10:** ANOVA table for material consumption.

Source	DF	Adj SS	Adj MS	F-Value	*p*-Value	Contribution (%)
Id	3	3.673	1.224	127.008	0.001	46.51
Mf	3	3.416	1.139	118.136	0.001	43.26
Wlc	3	0.698	0.233	24.145	0.013	8.84
Ip	3	0.080	0.027	2.764	0.213	1.01
Error	3	0.029	0.010			0.37
Total	15					100
R^2^ = 99.63%						

**Table 11:** Grey relational coefficient, grade, and rank.

Exp. No.	Grey Relational Coefficient GRC			Grey Relational Grade GRG	Rank
	Ts	Fs	Mc		
1	0.333	0.333	1.000	0.512	12
2	0.425	0.410	0.711	0.497	13
3	0.616	0.549	0.535	0.573	10
4	1.000	0.927	0.412	**0.820**	**1**
5	0.411	0.394	0.669	0.475	14
6	0.367	0.442	0.609	0.455	15
7	0.980	0.801	0.423	0.775	2
8	0.721	0.879	0.414	0.688	5
9	0.614	0.503	0.505	0.550	11
10	0.713	0.660	0.425	0.620	8
11	0.407	0.510	0.465	0.455	16
12	0.712	0.998	0.387	0.714	4
13	0.733	0.555	0.434	0.597	9
14	0.776	0.675	0.403	0.645	7
15	0.849	0.889	0.364	0.732	3
16	0.666	1.000	0.333	0.681	6

**Table 12:** Mean S/N ratios and ranking parameters of the GRG.

Level	Id	Mf	Wlc	Ip
1	−4.61	−5.49	−5.71	**−3.69**
2	−4.69	−5.22	−4.54	−5.16
3	−4.78	−4.15	−4.27	−4.70
4	**−3.58**	**−2.81**	**−3.14**	−4.12
Delta	1.19	2.68	2.57	1.47
∑ Delta	7.91			
Rank	4	1	2	3

**Table 13:** ANOVA table for GRG.

Source	DF	Adj SS	Adj MS	F-Value	*p*-Value	Contribution (%)
Id	3	0 015	0.005	2.099	0.279	7.37
Mf	3	0 092	0.031	12.848	0.032	45.10
Wlc	3	0.063	0.021	8.850	0.053	31.06
Ip	3	0.026	0.009	3.695	0.156	12.97
Error	3	0.007	0.002			3.51
Total	15					100
R^2^ = 99.49						

**Table 14:** Improvement in GRG.

	Initial Parameter Setting	Optimal Parameter	
		Prediction	Experimental
Setting level	Id_1_Mf_4_Wlc_4_Ip_4_	Id_4_Mf_4_Wlc_4_Ip_1_	Id_4_Mf_4_Wlc_4_Ip_1_
Ts (MPa)	12.98		13.58
Fs (MPa)	19.84		20.51
Mc (g)	4.73		5.46
GRG	0.820	0.919	0.894
Improvement in GRG = 0.074			
Percentage of improvement in GRG = 9.02%			

## Data Availability

The original contributions presented in this study are included in the article. Further inquiries can be directed to the corresponding author.

## Abbreviations

The following abbreviations are used in this manuscript:

AM	Additive manufacturing
ANOVA	Analysis of variance
DoE	Design of experiment
FFF	Fused filament fabrication
Fs	Flexural strength
GRA	Grey relational analysis
GRC	Grey relational coefficient
GRG	Grey relational grade
Id	Infill density
Ip	Infill pattern
Lh	Layer height
Mc	Material consumption
Mf	Material flow
PLA	Polylactic acid
SLA	Stereolithography
SLS	Selective laser sintering
S/N	Signal-to-noise
Ts	Tensile strength
UAV	Unmanned aerial vehicle
Wlc	Wall line count
